# Erosive Pustular Dermatosis of the Scalp with Urate-Like Crystals

**DOI:** 10.1155/2017/1536434

**Published:** 2017-10-08

**Authors:** Patrick O. Emanuel, Sharad P. Paul

**Affiliations:** ^1^University of Auckland, Auckland 1010, New Zealand; ^2^Auckland University of Technology, Auckland 1142, New Zealand; ^3^School of Medicine, University of Queensland, Brisbane, QLD 4006, Australia; ^4^Department of Surgery, University of Auckland, Auckland 1010, New Zealand

## Abstract

Follicular urate-like crystals were first described in Necrotizing Infundibular Crystalline Folliculitis (NICF), a rare cutaneous disorder with multiple waxy folliculocentric papules. Similar crystal accumulation may be seen within follicular infundibulae as an incidental finding. We describe a case showing identical crystals occurring within the horn-like crusts of a patient with erosive pustular dermatosis of the scalp (EPDS), a condition which due to its presentation can often be mistaken for nonmelanoma skin cancer. A brief overview of erosive pustular dermatosis of the scalp (EPDS) is presented in this paper.

## 1. Introduction

When a compound's supersaturation level exceeds the point of spontaneous crystallization, it can manifest as crystals on skin, as seen in calcium oxalate and oxalosis [1]. Follicular urate-like crystals were first described in Necrotizing Infundibular Crystalline Folliculitis (NICF), a rare cutaneous disorder with multiple waxy folliculocentric papules. Similar crystal accumulation may be seen within follicular infundibulae as an incidental finding. We describe a case showing identical crystals occurring within the horn-like crusts of a patient with erosive pustular dermatosis of the scalp (EPDS).

## 2. Case Presentation

**Figure 1:**
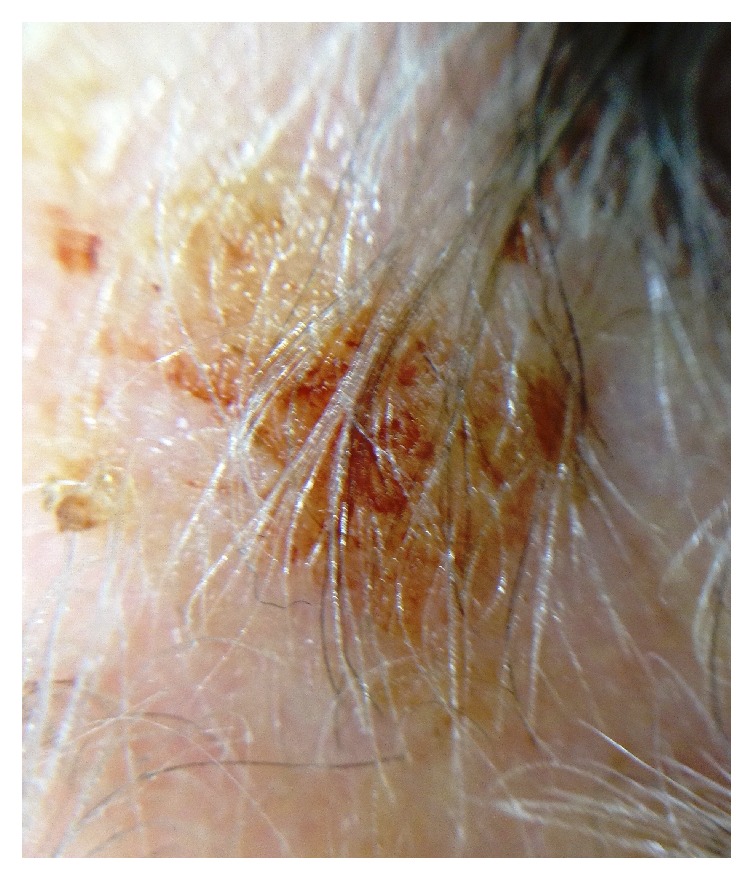
Clinical examination found multiple scalp erosions and crusted plaques.

An 81-year-old woman presented with erosions and crusted papules on the scalp and patchy areas of alopecia which had been progressive over many years. The largest of these was excised, as it was hyperkeratotic and ulcerated and therefore mistaken clinically for a squamous cell carcinoma. The rest of the scalp was covered with erosions and crusted plaques (Figure 1). Her past clinical history was significant only for numerous nonmelanoma skin carcinomas, especially squamous cell carcinomas on her scalp. Of note, there was no known history of hyperuricemia or gout. The patient presented with numerous actinic keratosis like areas, and in this case with a larger area that had ulcerated, and within the context of her history being one of actinic damaged scalp skin and multiple actinic keratoses, a diagnosis of squamous cell cancer was made.

**Figure 2:**
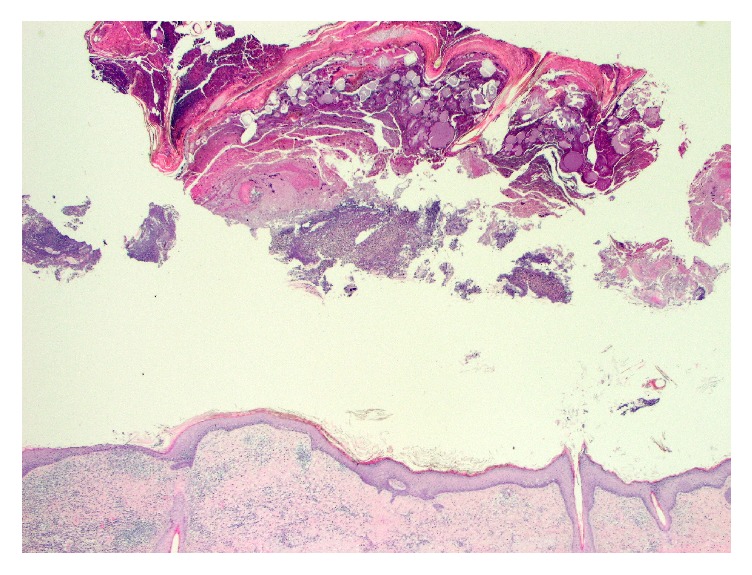
Excision revealed a variably atrophic epidermis with an impressive horn-like crust containing neutrophilic debris (H-E 2x).

**Figure 3:**
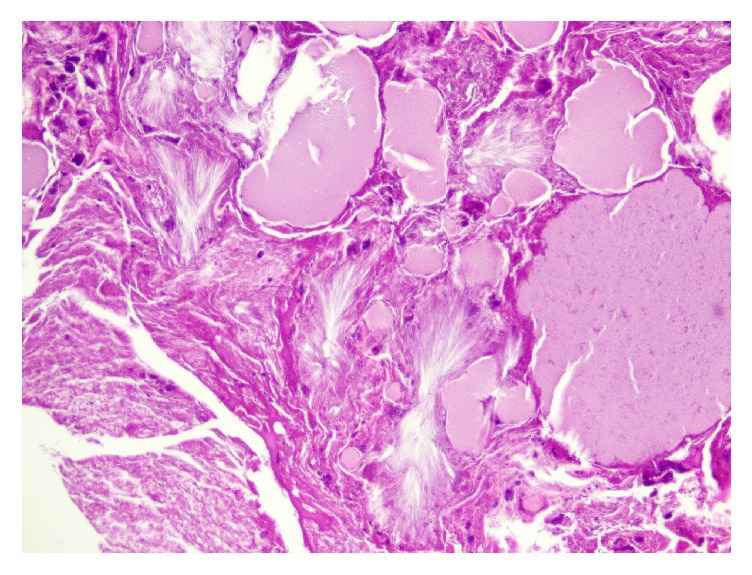
In areas, the material was revealed to have birefringent filamentous areas and a crystalline appearance (H-E 20x).

**Figure 4:**
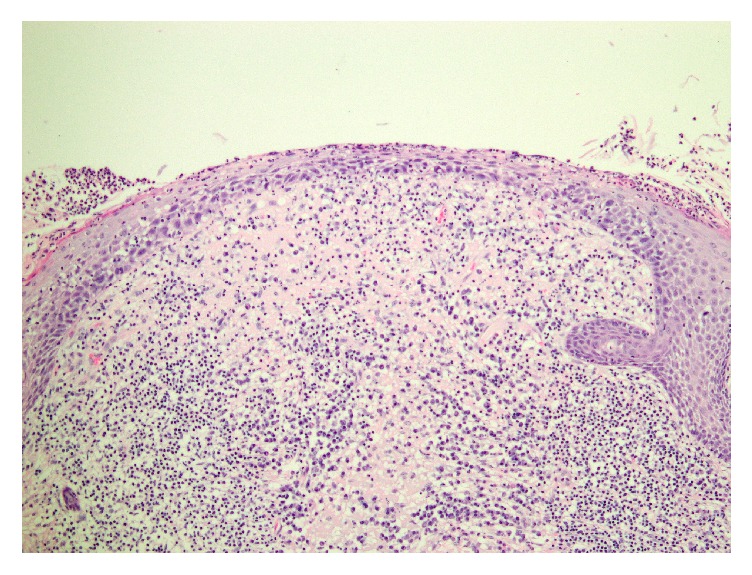
Neutrophils infiltrate the superficial layers of the epidermis. The dermis showed a mixed chronic inflammatory response and some dermal fibrosis.

Excision revealed a variably atrophic epidermis with an impressive crust containing neutrophilic debris (Figure 2). High power examination of the horn-like crust revealed numerous neutrophils, bacterial (coccal) colonies, and strikingly pale eosinophilic material (Figure 3). On close examination, this material was revealed to have birefringent filamentous areas and a crystalline appearance (Figure 3). Gram stain revealed gram-positive cocci in the crusted areas. PAS stain revealed rare pityrosporum species. In the epidermis, there was neutrophilic infiltration with accumulation in the superficial layers. The dermis showed a mixed chronic inflammatory response and some dermal fibrosis (Figure 4).

## 3. Therapeutic Intervention

The surgical scar healed well following the excision. We elected to treat this patient with a six-week course of oral minocycline 100 mg per day, and topical calcipotriol gel (calcipotriol gel at a concentration of 50 *μ*g/g) to the area and to adjacent lesions and this led to a complete clinical resolution of her scalp condition by the end of this period.

## 4. Discussion

We are presenting this case of erosive pustular dermatosis of the scalp (EPDS) as an underappreciated mimicker of skin cancer; the clinical picture can easily be mistaken for a keratinizing skin cancer or actinic keratoses.

The precise mechanisms of formation and composition of this crystalline material in NICF are currently unknown. Given the close morphologic resemblance to gout, it was initially hypothesized that the crystals were largely composed of urate. Given that most affected patients show no evidence of hyperuricemia or gout it was hypothesized that the urate may be related to something secreted by local microorganisms or cellular debris [1, 2]. Response to topical or systemic antibiotic treatments has been well documented in achieving a clinical response supporting the theory of bacterial involvement in the pathogenesis of formation of the crystalline material. By studying the material with electron microscopy, Lucke et al. [3] identified what were thought to be tonofilaments embedded in an amorphous matrix indicating that disruption of epidermal or follicular tonofilaments may play a role in the pathogenesis. Interestingly, urate-like crystals have also been observed in association with dermal xanthomas adding further support to the idea that a reaction with lipid or sebum-rich contents may also have the capacity to crystallize [4].

The etiology of EPDS is also poorly understood. Actinic damage and epidermal atrophy are thought to be a predisposing factor. Numerous other factors have been associated with the condition (various medications, infections, surgical procedures, or topical agents); it is unknown if they play a direct role in the pathogenesis of this condition. There have also been reports of this condition arising after topical treatment with ingenol mebutate [5]. Others have described this condition after photodynamic therapy [6].

It seems likely in the current case that the chronic inflammation and damage of the massive accumulation of keratin and inflammatory debris have contributed to the formation of the striking urate-like crystals. The patient had no history of prior use of urea-based creams or any other topical remedies that could have explained this presentation. To our knowledge, this finding of urate-like crystals in a case of erosive pustular dermatosis has never been reported previously, and therefore the case is being presented here.
